# Demographic and Spatial Predictors of Anemia in Women of Reproductive Age in Timor-Leste: Implications for Health Program Prioritization

**DOI:** 10.1371/journal.pone.0091252

**Published:** 2014-03-14

**Authors:** Andrew A. Lover, Mikael Hartman, Kee Seng Chia, David L. Heymann

**Affiliations:** 1 Saw Swee Hock School of Public Health, The National University of Singapore, Singapore; 2 Department of Surgery, The National University Hospital, Singapore; 3 Department of Medical Epidemiology and Biostatistics, Karolinska Institute, Stockholm, Sweden; 4 Public Health England, Department of Health, London, United Kingdom; 5 Department of Infectious Disease Epidemiology, The London School of Hygiene and Tropical Medicine, London, United Kingdom; Iranian Institute for Health Sciences Research, ACECR, Iran (Islamic Republic of)

## Abstract

Anemia is a significant risk factor for poor health outcomes for both the mother and neonate; however, the determinants of anemia in many epidemiological settings are poorly understood. Using a subset of a nationally representative cluster survey (2010 Demographic and Health Survey) in combination with other non-contemporaneous survey data, the epidemiology of anemia among women of reproductive age in Timor-Leste has been explored. Logistic regression was used to identify risk factors, population-level impacts were estimated as population attributable fractions and spatial analytics were used to identify regions of highest risk. The DHS survey found that ∼21% of adult women in Timor-Leste are anemic (49,053; 95% CI: 37,095 to 61,035), with hemoglobin <12.0 g/dL. In this population, the main risk factors (adjusted odds ratio; 95% CI) are: currently abstaining from sex for any reason (2.25; 1.50 to 3.38); illiteracy (2.04; 1.49 to 2.80); giving birth within the previous year (1.80; 1.29 to 2.51); consumption of fruits/vegetables low in vitamin A (1.57; 1.13 to 2.20); and the district-level confirmed malaria incidence (1.31; 1.15 to 1.49). A review of prior soil-transmitted helminth surveys in Timor-Leste indicates low-to-moderate prevalence with generally low egg counts, suggesting a limited impact on anemia in this setting, although comprehensive survey data are lacking. Examination of the population-level effects highlights the impacts of both recent births and malaria on anemia, with more limited impacts from diet; the evidence does not suggest a large contribution from geohelminths within Timor-Leste. These patterns are divergent from some other settings in the Asia-Pacific region and highlight the need for further focused research. Targeting high-burden districts and by increasing access to pre/postnatal care, raising literacy levels, increasing access to family planning, and improving malaria control should be prioritized to maximize inherently limited health budgets in reaching these populations.

## Introduction

Anemia is both a cause and an indicator of poor health states among women, neonates, and children. The primary risk factors globally are dietary deficiencies, high parity, diarrheal episodes, and parasitemia (malaria and geohelminths, especially hookworms) [1]. The World Health Organization (WHO) estimates that approximately 470 million non-pregnant women are anemic worldwide, with the largest contribution (39%) coming from the WHO Southeast Asian (SEARO) countries [2] [3]. Moreover, it has been estimated that anemia is the third largest contributor to total disability-adjusted life years (DALYs) among adult women (15–44 years) globally [4]. The etiology of anemia among women of reproductive age in developing countries is diverse, local and complex. The dominant risk factors in the Southeast Asia Pacific region overall are dietary iron deficiency, followed by malaria and high fertility, with more limited impacts from hookworms and HIV/AIDS [1] [5].

The 2010 Demographic and Health Survey (DHS) in Timor-Leste survey found that 21.3 percent of Timorese women aged 15–49 were anemic (17.5% mild, 3.6% moderate, <1% severe). Timor-Leste has several notable epidemiological factors: 100% of the country is at year-round malaria risk and the country had a total fertility rate of 5.7 in the three years prior to the survey, among the highest in the world outside sub-Saharan Africa [6] [7]. This unusually high birth rate, combined with limited access to health services in most areas contributes to high maternal (660 per 100,000 live births) and child mortalities [8]. Additionally, twenty-seven percent of women were found to be malnourished with BMI <18.5, indicating under-nutrition is a major health problem within Timor-Leste [7].

No prior analysis of risk factors for this demographic has been published although previous surveys in Timor-Leste suggest high prevalence of anemia throughout the country. The 2003 DHS found levels of 31.5% mild, 5.3% moderate and 1.0% severe anemia among non-pregnant women (aged 15–49); however, as no adjustment was made for smoking in this survey, the survey designers state that these levels cannot be compared with the 2010 survey [7] [9]. The aim of this research is to explore risk factors for anemia among women of reproductive age, to provide guidance for maximizing the impact of limited health resources in Timor-Leste.

## Methods

### Data sources

The 2009–2010 DHS was performed between August 2009 and January 2010 by the Timor-Leste National Statistics Directorate, in collaboration with ICF Macro (now ICF International; Calverton, Maryland, USA). It is the second large-scale demographic survey performed in the country; the first occurred in 2003. This survey utilized a standard DHS questionnaire that included several hundred queries to families, in addition to multiple country-specific modules.

Hemoglobin levels were measured via the HemoCue system and were categorized by the DHS as mild, (10.0 to 11.9 g/dL), moderate (7.0 to 9.9 g/dL), or severe anemia (<7.0 g/dL), with “any anemia” corresponding to <12.0; all Hb values were corrected by the survey designers for both altitude and smoking status [7] [10]. While these subclasses differ slightly from updated WHO standards, the primary outcome of anemia (Hb<12 g/dL) is consistent [11].

The total female population of reproductive age (ages 15–49) for each district was compiled from the 2010 Timor-Leste National Census [12]. District level malaria data was obtained from the 2007 Ministry of Health summary, and consists of reported clinical, confirmed and total cases per 1000 population from 2006; this is the most recent data publicly available at this reporting level [13].

To address the lack of soil-transmitted helminth (STH) data within the DHS, we performed a limited systematic review of STH prevalence in Timor-Leste. A literature search was performed in PubMed and Google Scholar, using the search query (“Timor” AND (“helminth” OR “hookworm” OR “*Ascaris*” OR “*Trichuris*” OR “*Strongyloides*” OR “*Ancylostoma*” OR “*Necator*”)). The bibliographies of identified papers were then consulted, and other sources identified.

### Sampling plan

**Figure 1:**
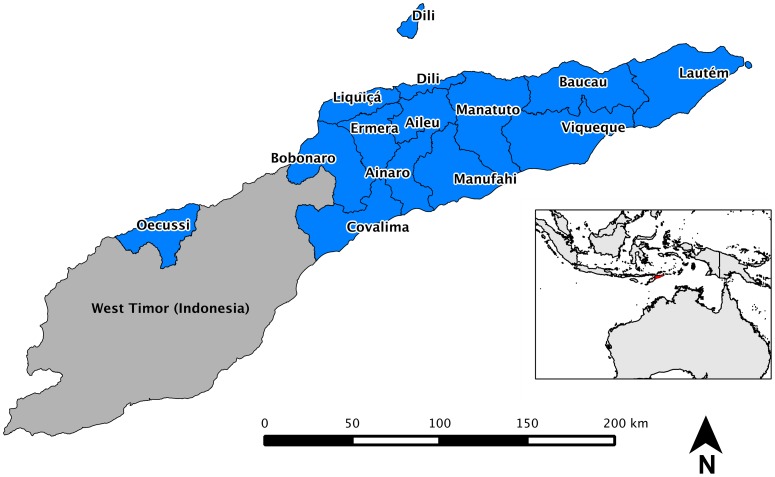
Overview of Timor-Leste.

The clusters selected by ICF Macro include independent samples from two strata (rural and urban) in all thirteen districts throughout the country (figure 1), with sampling based upon the 2004 Timor-Leste Population and Housing Census [7].

In total, 12,285 households were selected for sampling, and approximately 11,800 interviews of women aged 15–49 were completed [7]. Of these, a subset of 4,059 women was selected to have hemoglobin levels captured; of this group, 1,527 were included in the dietary sub-sections of the survey. For our study, pregnant women and those with multiple missing predictors or data inconsistencies were removed, leaving 1,277 women. Observations with outlying covariate patterns were then removed, to give a final sample of 1,255 women.

The spatial data was provided by ICF Macro as an Esri shapefile and includes a central coordinate for each survey cluster, but households were not individually reported for privacy, and some clusters have had their coordinates displaced by 0 to 10 km (rural) and 0 to 2 km (urban) [7].

### Data analysis: regressions

Initial univariate analysis to verify an association with anemia utilized all explanatory variables identified in similar studies and those with biological plausibility. All tests were two-tailed, and all models incorporated adjustment for the complex cluster sampling design.

To verify an association between categorical variables, the **χ^2^** test was employed; if a cell had an expected frequency below 5, Fisher's exact test was used. For each investigated variable, the odds ratio was calculated using univariate logistic regression, and all exposure variables with a p-value below 0.2 were included in the starting variable set. Akaike and Bayesian Information Criterion (AIC/BIC) were used as primary drivers for model building, and models checked with the Archer-Lemeshow goodness-of-fit test (by deciles) [14]. Receiver-operating curve (ROC) plots and sensitivity/specificity tables were also utilized to assess the practical utility of the individual models. After identification of a preliminary refined model, fractional polynomial analysis was assessed for the continuous variables; these showed only modest improvement in model fit, at the cost of a model that could not be intuitively evaluated. Confounding was also assessed: case-control tabulation and bivariate regression analyses were used to examine potential interactions (defined as >15% change in coefficient of the referent variable). Residuals were examined and a population of highly influential observations was identified and removed from the analysis.

The odds ratios for interventionable risk factors were then used to calculate the population attributable risk fractions [15]. To estimate the total number of anemic adult women nationally and by district with associated confidence intervals, the survey sampling errors reported by ICF Macro were combined with data from the 2010 Timor-Leste National Census [7] [12]. Statistical analyses were performed using Stata 11.2 (College Station, Texas, USA). Calculation of the population attributable fraction utilized the Stata - punaf - program [16].

### Data analysis: spatial

Initial spatial analysis involved linking the reported anemia prevalence (as proportions) for each cluster to the corresponding geospatial location (survey cluster values). Pooled anemia percentages were then computed for both districts and sub-districts in Stata, and these values were imported into the QGIS data structure. Spatial interpolation was performed using inverse distance weighting [17]. Spatial analyses were performed using QGIS Version 1.5.0 (“Copiapo”) and base files of the administrative districts for Timor-Leste were obtained from maplibrary.org and the Timor-Leste National GIS Portal [18].

### Ethics review

The 2010 DHS in Timor-Leste received ethics approval from the Timor-Leste Ministry of Health, and the anonymized study datasets are freely available for the DHS at www.measuredhs.com.

## Results

### Risk factors for anemia

The study population was a subset of 1,255 women from the total national survey; the characteristics can be found in Table 1. Noteworthy features are many recent births, low population BMI, and an overwhelmingly rural population; these values are consistent with the full survey population [7].

**Table 1:** Characteristics of study population among women of reproductive age, Timor-Leste (BMI- body mass index; Hb- hemoglobin; SD- standard deviation).

Characteristic			N, (%)
Age	15–19		56 (4.5)
	20–24		247 (19.7)
	25–29		302 (24.0)
	30–34		237 (18.9)
	35–39		244 (19.4)
	40–44		128 (10.2)
	45–49		41 (3.3)
Rural			973 (77.5)
Literate	Yes or partially		798 (63.6)
	No		457 (36.4)
Education	None		412 (32.8)
	Primary		372 (29.6)
	Pre-secondary		215 (17.1)
	Secondary		230 (18.3)
	Higher		26 (2.1)
Bed net usage, previous night	None		629 (50.1)
	Treated net		581 (46.3)
	Untreated net		45 (3.6)
Births in previous 1 year	Zero	(age <40)	549 (50.6)
		(age 40–49)	112 (66.3)
	1–2	(age <40)	537 (49.4)
		(age 40–49)	57 (33.7)
Smoker			51 (4.1)
Currently abstaining from sex for any reason			247 (19.7)
Mean BMI (SD)			20.2 (2.57)
Low BMI (<18.5 kg/m^2^)			332 (26.5)
Mean Hb (g/dL), corrected (SD)			12.8 (1.43)
Anemia	None (Hb>12.0 g/dL)		982 (78.3)
	Mild (Hb 10.0 to 11.9 g/dL)		221 (17.6)
	Moderate (Hb 7.0 to 9.9 g/dL)		48 (3.8)
	Severe (Hb<7.0 g/dL)		4 (0.3)
			(N = 1255)

The relationships between risk factors and anemia from multivariate logistic analysis are shown in Table 2. The main risk factors for anemia in this population are currently abstaining from sex for any reason (adjusted odds ratio = 2.25; 95% CI 1.50 to 3.38); and illiteracy (OR = 2.04; 95% 1.49 to 2.80). It is important to note that abstinence from sex in Timor-Leste is generally associated with the postnatal period, and essentially all Timorese women in the overall DHS who recently gave birth (93 percent) are still abstaining from sex in the eight weeks after giving birth [7]. While the survey did not differentiate between this trend and any other reasons for abstinence from sex, the interaction between “currently abstaining from sex” and “giving birth in the previous year” was not significant in the multivariate analysis (p = 0.67). Giving birth within the previous year (OR = 1.80; 95% CI 1.29 to 2.51) was also an independent risk factor for anemia.

**Table 2:** Risk factors for anemia (Hb<12.0 g/dL) among women of reproductive age Timor-Leste from logistic regression models.

Covariate		Univariate Odds Ratio	95% CI	p value	Adjusted Multivariate Odds Ratio	95% CI	p value
Currently abstaining from sex for any reason (see discussion)	No	Ref.			Ref.	-	
	Yes	2.61	1.82 to 3.74	<0.001	2.25	1.50 to 3.38	<0.001
Illiteracy	No	Ref.			Ref.	-	
	Yes or partial	1.75	1.30 to 2.36	<0.001	2.04	1.49 to 2.80	<0.001
Births in Previous 1 Year	Zero	Ref.			Ref.	-	
	1–2	2.08	1.53 to 2.81	<0.001	1.80	1.29 to 2.51	0.001
Other fruits, night before survey		1.35	1.00 to 1.84	0.052	1.57	1.13 to 2.20	0.009
District-level confirmed malaria rate per 1000 population (2006)	1.1 per 1000	Ref.			Ref.		
		1.23	1.10 to 1.37	<0.001	1.31	1.15 to 1.49	<0.001
BMI, continuous		1.00	0.999 to 1.00	0.011	1.00	0.998 to 1.00	0.002
Height, cm		1.00	0.992 to 0.998	0.001	0.99	0.991 to 0.996	<0.001
Cluster altitude		1.00	1.00 to 1.00	0.382	1.00	1.00 to 1.00	0.013

**Figure 2:**
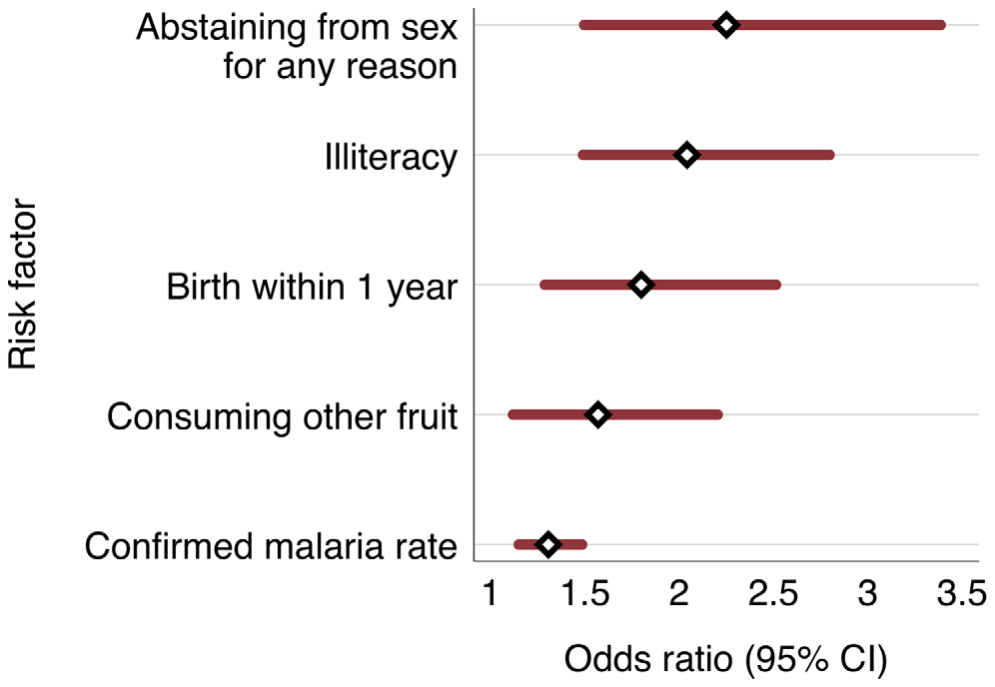
Adjusted odds ratios, risk factors for anemia among women of reproductive age in Timor-Leste. (Confirmed malaria rate is 2006 district level value per 1000 population).

Consumption of “other vegetables,” (defined within this survey as any fruit or vegetable *other* than high vitamin-A fruits like pumpkin, squash, carrots, sweet potatoes, green leafy vegetables, mangoes, or papayas) was associated with higher anemia risk (OR = 1.57; 95% CI 1.13 to 2.20). Finally, the district-wide confirmed malaria (modeled as the logarithm of the continuous reported value) showed an association with anemia (OR = 1.31; 95% CI 1.15 to 1.49). That is, the odds of anemia increase by 30% for each 10-fold increase in the confirmed malaria rate. The range in confirmed malaria rate (actually a ratio, per 1000) ranges from 1.1 to 151.3 for 2006 in the thirteen districts. Figure 2 illustrates the relative magnitude of these risk factors with the associated 95% confidence intervals.

### Population at risk and attributable fractions

**Figure 3:**
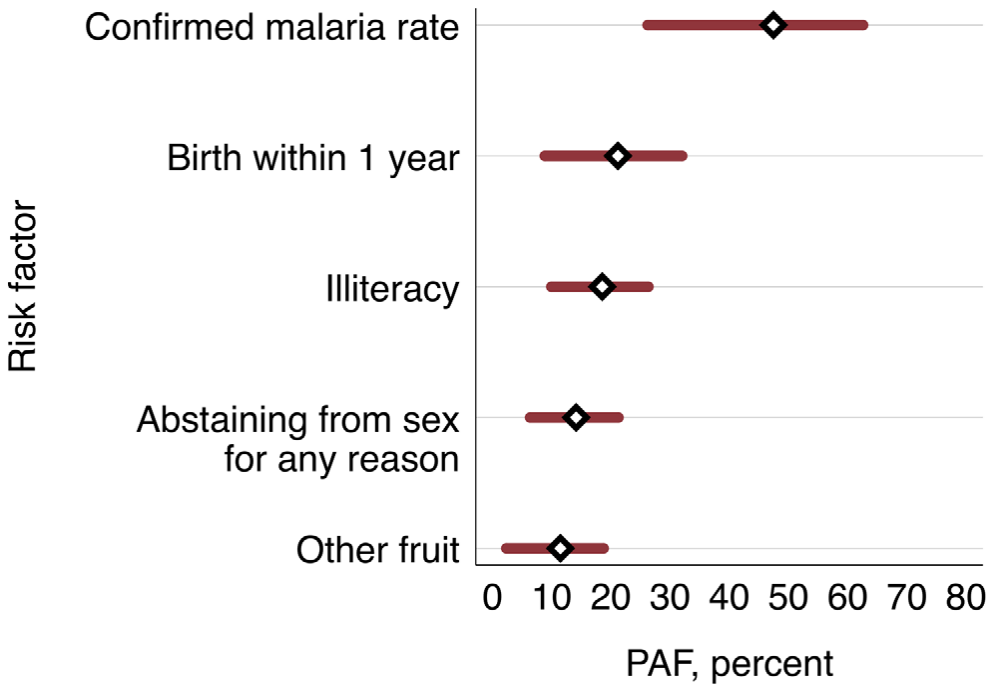
Population attributable fractions for anemia among women of reproductive age in Timor-Leste (see Table 3 for reference groups).

The population attributable fraction for each of the interventional significant risk factors can be seen in table 3, and are compared in figure 3. The district wide confirmed malaria rate was the largest contributor, with 47.5% of the burden (95% CI 26.2 to 62.6, with 1.1 per 1000 as the reference category). For the other factors, although there is overlap of the confidence intervals, the lower bounds of the 95% CI differ and therefor give the *minimum* contribution to the population anemia. Illiteracy was the second most significant factor at 18.5% (95% CI 9.9 to 26.3; full or partial literacy as reference), followed by giving birth within the previous year, 21.2% (8.8 to 32.0; not giving birth as reference). The next most significant factor identified is currently abstaining from sex for any reason with 14.1% of the burden (95% CI 6.4 to 21.3; not abstaining as reference). Lastly, the impact of consuming other fruit as defined above, is 11.5% (95% CI 2.3 to 18.8; consuming fruit high in vitamin A as reference).

**Table 3:** Population attributable fractions for modifiable risk factors for anemia among for women of reproductive age in Timor-Leste.

Risk Factor	Comparison group	PAF, %	Range (95% CI)
Confirmed Malaria Rate, per 1000	1.1 per 1000 (Ref.)	47.5	26.2 to 62.6
Giving birth within 1 year	Not giving birth (Ref.)	21.2	8.8 to 32.0
Illiteracy	Full or partial literacy (Ref.)	18.5	9.9 to 26.3
Abstaining from sex for any reason (see text)	Not abstaining (Ref.)	14.1	6.4 to 21.3
Consuming other fruit (see text)	Not consuming other fruit (Ref.)	11.5	2.3 to 18.8

Note: PAF  =  Defined as [p(aOR - 1) / aOR] where p is proportion of cases of anemia exposed to a risk factor, and aOR is the adjusted odds ratio from logistic regression analysis [15].

Combination of national census data (2010) and the anemia prevalence by districts from the DHS survey allowed us to estimate the total population at risk throughout the country with appropriate confidence intervals: 49,053 (95% CI: 37,095 to 61,035), with a large number 10,361 (95% CI: 7,755 to 12,967) of these residing in the Dili district. The district-specific values can be found in table 4.

**Table 4:** Estimated total female anemic population, Timor-Leste.

District	Prevalence, anemia, % (95% CI)	Population, female age 15–49	Estimated total number anemic (95% CI)	Estimated % of national burden (95% CI)
Dili	16.7 (12.5 to 20.9)	62,042	10361 (7755 to 12967)	21.1 (20.8 to 21.5)
Ermera	21.4 (16.8 to 26.0)	25,408	5437 (4269 to 6606)	11.1 (10.8 to 11.4)
Bobonaro	25.6 (19.8 to 31.4)	20,194	5170 (3998 to 6341)	10.5 (10.3 to 10.8)
Baucau	17.3 (12.0 to 22.5)	23,006	3980 (2761 to 5176)	8.1 (7.9 to 8.4)
Viqueque	25.2 (20.7 to 29.7)	14,106	3555 (2920 to 4189)	7.2 (7.0 to 7.5)
Covalima	25.4 (19.8 to 31.1)	13,127	3334 (2599 to 4082)	6.8 (6.6 to 7.0)
Oecussi	22.5 (17.2 to 27.9)	14,414	3243 (2479 to 4022)	6.6 (6.4 to 6.8)
Lautem	25.5 (20.2 to 30.8)	12,301	3137 (2485 to 3789)	6.4 (6.2 to 6.7)
Liquiçá	20.7 (15.4 to 26.0)	14,159	2931 (2180 to 3681)	6.0 (5.8 to 6.2)
Manatuto	32.6 (25.3 to 39.9)	8,728	2845 (2208 to 3482)	5.8 (5.6 to 6.0)
Aileu	27.5 (21.2 to 33.9)	9,500	2613 (2014 to 3221)	5.3 (5.1 to 5.5)
Manufahi	12.8 (8.6 to 17.1)	10,006	1281 (861 to 1711)	2.6 (2.5 to 2.8)
Ainaro	10.1 (4.9 to 15.3)	11,553	1167 (566 to 1768)	2.4 (2.2 to 2.5)
National Total		238,544	49,053 (37,095 to 61,035)	-

### Spatial analysis

**Figure 4:**
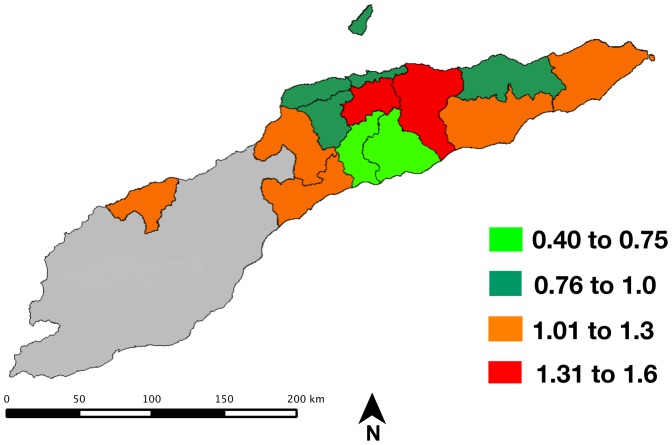
Standardized prevalence ratio for anemia among women of reproductive age in Timor-Leste (normalized to national prevalence of 23.1%).

**Figure 5:**
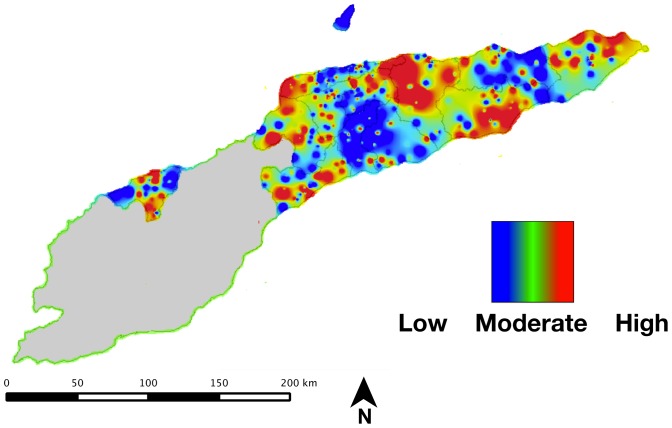
Smoothed anemia prevalence for anemia among women of reproductive age in Timor-Leste (range = 10.1 to 32.6%).

Our spatial analysis of anemia in Timor-Leste relies on multiple analyses and visualizations to provide a comprehensive view of the spatial variation in risk. The rates of anemia are compared across districts in figures 4 and 5.

The standardized prevalence ratios by district (normalized to the national mean prevalence of 21.3%) ranged from 0.4 to 1.56; the overlay of these values is shown in figure 4. Two districts show elevated prevalence - Aileau (1.31) Manatuto (1.53). Women in these districts have a 31% and 53% greater risk respectively of being anemic relative to the national mean; several districts show markedly lower prevalence - Ainaro (0.47) and Manufahi (0.60).

The smoothed national anemia prevalence as measured in the survey is shown in

Figure 5. This map shows the national values interpolated from the individual survey cluster values, and provides an estimate of the anemia prevalence in areas not captured in the national DHS.

### Review of soil-transmitted helminth (STH) surveys in Timor-Leste

No direct data on STMs was recorded during the DHS survey; a proxy question of “Any drugs for intestinal parasites in the previous six months” was not significant in the final model (14.6% of the 1,255 women reported “yes”). To address this limitation, we incorporated a systematic review of soil-transmitted helminth surveys in Timor-Leste. The only survey to specifically target women of reproductive age was fielded in 2006, and examined 466 persons in three villages in western Timor-Leste (Lautem and Covalima districts, see figure 1), which included all residents of the villages aged five years and older. The overall prevalence of hookworm, *A. lumbricoides* and *T. trichiura* was 34.8%, 1.3% and 0.9% respectively; essentially all hookworm infections were light (97.2%) [19]. The prevalence of hookworm in this survey among adult women specifically was 36.1%, and all of these were light infections by standard WHO criteria. A recent cross-country comparison supports this view, and rates hookworms as a “minor” health problem among adult women in Timor-Leste [20].

There is a general correlation between prevalence of STHs among children and adults within communities [21]. Surveys among children in Timor-Leste have also found limited prevalence: a 2002 study found moderate levels of helminths in central Timor-Leste: from 13.6 to 75.6% of children were positive for *A. lumbricoides*; however, no hookworm was identified [22]. A nationally representative survey of children aged 7 to 16 performed in 2012 did not quantify egg burden, but found a national STH prevalence 29.0%, ranging from a low of 4% in Manatuto district to a high of 55% in Dili district, with a statistically significant decrease by age; the national prevalence of hookworm in this survey was 9.2% (95% CI: 8.0 to 10.0) [23].

## Discussion

Our results suggest among women of reproductive age in Timor-Leste that malaria and high fertility rates are more important causes of anemia than dietary factors or illiteracy, and limited evidence suggests a smaller contribution from helminths.

The prevalence of anemia in Timor-Leste is extensive - we estimate that 49,053 (95% CI: 37,095 to 61,035) adult women are anemic. These estimates are aligned with prior work from the United Nations, which in 2005 estimated that about 55,000 [95% CI: 51,000 to 59,000] women of reproductive age were anemic in Timor-Leste [15]. However, our estimates allow district-specific comparisons to be made (table 4).

Several regions within Timor-Leste show elevated risk when examined as aggregate values and indicate that several districts have a significant proportion of the national burden in Timor-Leste. Specifically, Dili, Ermera and Bobonaro districts have very high prevalence of disease relative to other regions (figures 1 and 2).

Within the Southeast Asia region, dietary factors have been reported to be major contributors to anemia: the dominant factor in the region overall has been reported to be dietary iron deficiency, followed by malaria and high fertility, with hookworm and HIV/AIDS having more limited impacts [1]. For example, a cross-sectional national survey from Vietnam found that hookworm infection, climatic zone, consumption of less than one serving of meat per week, having more than three children, and having a child under 24 months old all had a significant association with hemoglobin levels in women of reproductive age [24].

Our results suggest that Timor-Leste diverges from these regional trends: while diet does show some impact, the effect is dwarfed by reproductive factors and malaria. Moreover, as the HIV/AIDS prevalence in Timor-Leste has been reported as <0.2%, it is not considered a major contributor to anemia [25]. The most comprehensive published analysis to date of anemia in Timor-Leste examined risk factors among 4,514 children aged 6-59 months. The primary risk factors identified in this study population were household wealth, dietary insufficiency (wasting), diarrheal episodes, maternal education, and maternal anemia status [26].

Population attributable fractions allow ranking of potential interventions to maximize impact. This metric is a measure of the total effect of the risk factor on population anemia; that is, how much of the population-level outcome would be addressed if the exposure could be eliminated entirely. Within this framework, malaria control would be expected to have the largest impact on anemia in women of reproductive age in Timor-Leste.

Surprisingly, the use of bed nets (treated or otherwise), a key malaria intervention, on the prior night was not correlated with anemia within this survey. However, this is likely the result of low overall usage as measured by the same survey: for the night prior to survey, only 41.4% of urban and 31.7% rural women reported sleeping under an insecticide treated net. Additionally, these reported values might overstate proper usage, as demonstrated by several studies in the region [27] [28], and bed net usage in Timor-Leste may be compromised by complex economic and social decisions within households [29].

In other settings, the population attributable fraction (PAF) contribution from malaria has been highly variable. Our results are consistent with those from a study in Thailand that identified malaria during pregnancy as the largest contributor to anemia, with a PAF of 27% (95% CI: 16 to 30); which remained significant at delivery with a PAF of 17% (95% CI: 9 to 23) [30]. A nutrition-specific study from Tanzania found an anemia PAF of 12% (no 95% CIs presented) from malaria in ages >15, with a total parasite prevalence of 19.6%, and was identified as the largest contributor to anemia burden in the study [31]. In contrast, a study among pregnant women a low transmission setting in Nepal identified *Plasmodium vivax* parasitemia as having more limited impact, with a PAF of 5% (no 95% CIs presented) of all anemia [32].

The survey in Timor-Leste was fielded from mid-August 2009 to early February 2010, and captured much of the malaria high-transmission season (November to May). The use of ecological malaria indexes is likely one cause of the higher PAF in Timor-Leste, and should be interpreted cautiously. However, this larger impact of malaria on anemia in Timor-Leste could also be explained by the prevalence of *Plasmodium vivax*, plus *P. falciparum / P. vivax* co-infections within the population. *P. vivax* infection has significant hematological impacts, and co-infection with these parasites has been associated with more severe anemia relative to infection by individual species in Indonesian Papua and Papua New Guinea [33] [34]. Moreover, a strong relationship has also been documented between population-level anemia and malaria transmission indexes in Papua New Guinea, supporting a prominent role for malaria in the observed patterns of anemia in Timor-Leste [35]. Other infectious diseases, including tuberculosis or other recent bacterial/viral infections were not directly queried in the survey and therefore it is not possible to assess their impact on measured anemia, but their potential role should be explored in future surveys.

The very large impact of high fertility is a striking feature of the situation in Timor-Leste, and mirrors findings in high fertility countries in sub-Saharan Africa.

The positive association with currently abstaining from sex for any reason appears counter-intuitive; however, in Timor-Leste, there is a cultural norm of women abstaining for several months after the birth of a child, but the survey data does not distinguish this from other reasons [7]. Future qualitative and quantitative work should be used to more fully understand this complex metric. The impacts of high fertility and a limited health sector can be inferred from several indices in Timor-Leste. The TFR (total fertility rate) in this survey was found to be 6.0 (4.9 urban, and 5.7 rural), which is the highest in the world outside of sub-Saharan Africa and the maternal mortality rate has been estimated to be 557 (95% CI 408 to 796) per 100,000 [7]. One contributor to these high indexes is that only 29.9% of births occur with skilled assistance (59.1% rural and 20.7% rural), and the timing of births is also noteworthy with many closely spaced pregnancies −8.9% of women reported a birth interval of 7–17 months, and 20.3% reported an interval of 18–23 months [7]. While there is reasonable uptake of antenatal services (55% of pregnant women in the survey made four or more antenatal clinic visits) the high levels of anemia in women of reproductive age suggest missed opportunities for iron supplementation.

The combination of these factors likely elevates reproductive elements to such a prominent risk factor among women in Timor-Leste. The biological mechanisms that underlie the high impacts of births within the prior year and abstinence from sex for any reason are likely a multi-factorial combination of increased iron demands during pregnancy, higher susceptibility and impact of malaria and other infectious diseases during pregnancy, and blood loss during delivery [36]. However, the respective contributions of these interconnected factors in this population cannot be assessed from these survey data.

Micronutrient deficiency (especially iron, folic acid, iodine, zinc, and vitamins A and D) is also considered a major contributor to anemia [37]; however, we find only a moderate association and the population attributable fraction is limited. A single query regarding consumption of vitamin A containing fruit was significant in the multivariate analysis; all other self-reported dietary questions on micro - and macronutrients were not associated with anemia status.

There are several plausible reasons for this finding. Foremost, the dietary survey queries are limited in scope, and may therefor be unable to capture daily or seasonal variations in diet. Alternatively, the overall nutritional status of women in Timor-Leste could be precarious enough that the differential among anemic and non-anemic women is limited. This interpretation is congruent with anthropometry captured within the same overall DHS: 27% of women were malnourished with a BMI <18.5; and 53% of children were classified as stunted, with 17% classified as wasted. All of these suggest low population levels of sufficient nutrition in Timor-Leste, and may explain the limited impact of the surveyed nutritional factors.

The impact of illiteracy on the risk of anemia may be due to limited ability to seek care. This interpretation is supported by measured empowerment within the same survey - a small minority (22.8%) of married women reported being able to make their own decisions about their health care; however, due to very limited overlap of anemia testing and the empowerment module in the survey, we were unable to include these factors in our analysis. A similar impact from illiteracy and subsequent limited empowerment has been identified in some other studies, including one in India [38] but other studies have not found a significant effect of illiteracy among women of reproductive age [39].

Studies in a wide range of transmission settings have found that prevalence of STH infections peaks in school-age children and then tapers into adulthood; the intensity of infection (mean worm burden) shows the same general trends, except for hookworm, where intensity may peak in early adulthood [21]. However, other populations have shown no differences in worm burden among anemic and healthy subjects [40], and several studies have shown that only very high hookworm burdens are associated with anemia [41] [42]. A study in Thailand found only a marginally significant independent association between density high hookworm infections (≥1,000 ova/mL) and anemia among pregnant women with an adjusted odds ratio of 2.05 (95% CI: 1.01 to 4.20; p = 0.049); no association with low density infections was found in multivariate analysis [43].

While none of the surveys we have identified were specifically designed to address the impact of STHs on anemia in Timor-Leste, taken together these observations suggest that geo-helminths are not a major contributing factor to the burden of anemia among women of reproductive age within Timor-Leste. However, as comprehensive, temporally aligned data do not exist, this conclusion should be considered speculative until detailed surveys are fielded.

The spatial contrasts in anemia prevalence highlighted here have multiple possible interpretations. The risk of anemia could vary due to regional differences in infectious disease risk; dietary preferences or nutritional insufficiency; or access to care. The two districts with the highest standardized ratio occur in the higher elevation regions of the country; however, as the hemoglobin levels have been adjusted for altitude, an independent effect is likely.

There have been only limited reports on the spatial variation of anemia risk. In Angola, a spatial model of anemia among children found that malnutrition and parasitological risk factors were highly heterogeneous and contributed to the spatial variation in individual-level anemia risk [44]; these factors are likely also important in Timor-Leste. A second possible explanation is that the observed differences are due to aggregation of data across either survey clusters or districts. This effect, termed the “modifiable areal unit” has the potential to produce biased effect measures, and needs to be considered in the reporting of aggregated disease data [17]. The trends we have identified in spatial variation should be considered exploratory until further surveys are performed.

### Strengths and Limitations

The stringent cluster sampling and comprehensive national sampling frame suggests that these data are representative of the overall population experience. However, there are several inherent limitations. First, the cross-sectional nature of the survey does not allow causality to be unambiguously determined; secondly, dietary questions queried only what was eaten the previous night, and may not fully represent the daily or seasonal diet. A third limitation is that the malaria data are annual aggregate ratios from districts, and not temporally aligned with the survey. While clearly ecological, these values still serve as a proxy for the force of infection at a population-level, but do not represent individual-level disease risk. Finally, we were also unable to rigorously assess in regression analysis the impact of soil-transmitted helminths due to a lack of integrated data sources.

### Conclusions: Implications for Health Programs

The results from this study suggest several paths whereby inherently limited funds for interventions can be maximized in terms of health benefit. Pregnant women and women with recent births, those with lower literacy, and women living in areas of high malaria transmission should be prioritized in any intervention program. Subsequent surveys should also attempt to address the malaria epidemiology to potentially allow greater delineation of co-epidemiology with anemia, potentially via a Malaria Indicator Survey.

The districts with the greatest numbers of anemic women (Dili, Ermera, Bobonaro) should be prioritized, as they account for more than 40% of the national burden. The PAFs suggest that priority interventions should include malaria control and expanded pre/postnatal care. These results reinforce the complex multi-factorial nature of anemia in developing countries, and the pressing need for integrated, cross-sector programs that prioritize infectious disease, birth spacing, food security, and education to comprehensively address this issue [45] [46]. Strong evidence exists to support population-level malaria control, dietary supplementation, and prenatal iron supplementation to drive impact towards the Millennium Development Goals [47] [37] [3]. Moreover, prioritization and integration of these programs would have broader health impacts in other vulnerable sub-populations including neonates, pregnant women and children under-five [48], as adult women's health status and risk factors for anemia likely reflect underlying interrelated issues within these broader populations. These interconnections have been highlighted as key health research priorities within Timor-Leste [49]; however, large gaps remain in understanding the underlying causes of anemia, and more research is needed to elucidate the complex interplay between dietary, social and infectious disease factors within Timor-Leste and throughout Southeast Asia.
